# Indoor Tracking to Understand Physical Activity and Sedentary Behaviour: Exploratory Study in UK Office Buildings

**DOI:** 10.1371/journal.pone.0127688

**Published:** 2015-05-20

**Authors:** Richard Spinney, Lee Smith, Marcella Ucci, Abigail Fisher, Marina Konstantatou, Alexia Sawyer, Jane Wardle, Alexi Marmot

**Affiliations:** 1 UCL Institute of Environmental Design and Engineering, Bartlett Faculty of the Built Environment, UCL, Central House, 14 Upper Woburn Place, London, WC1H 0NN, United Kingdom; 2 Health Behavior Research Centre, Department of Epidemiology and Public Health, UCL, 1–19 Torrington Place, London, WC1E 7HB, United Kingdom; Columbia University, UNITED STATES

## Abstract

Little is known of the patterns of physical activity, standing and sitting by office workers. However, insight into these behaviours is of growing interest, notably in regard to public health priorities to reduce non-communicable disease risk factors associated with high levels of sitting time and low levels of physical activity. With the advent and increasing availability of indoor tracking systems it is now becoming possible to build detailed pictures of the usage of indoor spaces. This paper reports initial results of indoor tracking used in conjunction with the ActivPAL activity monitoring device. In this paper we give an overview of the usage of the tracking system and its installation and illustrate some of the resultant data. We also provide preliminary results that investigate the relationship between location, light physical activity and sitting in a small sample of office workers (n=33) from two separate office environments in order to demonstrate the relevance and explanatory power of the technique.

## Introduction

Regular participation in physical activity (PA) is known to reduce several non-communicable disease risk factors [1]. An emerging body of literature suggests that prolonged bouts of sedentary time (i.e. sitting time [ST]) is associated with higher risk of cardiovascular disease and mortality, even after statistical adjustment for moderate-to-vigorous physical activity (MVPA; e.g., brisk walking) [2]. Some data also suggest that interruptions in prolonged periods of ST are beneficially associated with metabolic health [3]. However, current PA levels in adult populations have been found to be low in several countries [4], and in advanced economies a large proportion of adults of working age have sedentary office jobs [5].

In a study of desk-based workers it was found that ‘work time’ was associated with more ST and less PA than ‘non-work’ time, the study also found that the workplace is a key setting for prolonged ST [6]. Two reviews identified that PA promotion strategies can be effective at increasing PA among desk-based workers [7, 8]. However, they identified that most interventions have focused on psychological and social determinants and have typically produced small effects. A recent review on reducing ST in office buildings found that interventions predominantly targeted the individual and were often unsuccessful [9], though more recent interventions have achieved reductions in sitting [10–13]. Whilst some interventions have specifically targeted greater use of stairs [14], we are not aware of any studies that are implemented or designed based on an understanding, in fine detail, of where and how PA and ST are accumulated in desk-based environments. However, some research has suggested that indoor factors such as the number, distribution and density of office destinations could have an impact on desk-based workers’ PA/ST [15]. For an understanding of any such relationship to be properly understood or effectively exploited through intervention it is desirable to have a clear picture of the PA/ST generation in these locations and how it occurs. However, currently, this picture is not available to researchers.

In contrast to indoor environments, PA, and the location of its accumulation, in the outdoor environment has been more extensively studied, using global positioning system (GPS) technology, often alongside Geographic Information Systems (GIS) [14]. However, GPS technology cannot be used within buildings since the satellite signals are blocked by their physical structures. Indoor tracking systems [15] are a potential alternative solution. “Indoor tracking” is an umbrella term for several techniques and technologies used to monitor location and movement within buildings with several different existing approaches [15–18]. Common approaches use technologies such as radio-frequency-identification (RFID), wireless local area networks (WLAN) and Bluetooth to determine location through techniques including triangulation and direct proximity inference[19]. An output in the form of time-stamped information about the location of the person or object being monitored characterises all approaches, however the resolution, accuracy, format and nature of location information can vary dramatically [20].

In this paper we present a novel application that combines indoor tracking using a time-stamped location of monitored individuals, with time-stamped accelerometer-based measurements to determine where and how sitting, standing and stepping behaviours occur. The paper gives an overview of: i) the tracking and accelerometer technologies deployed; ii) how they are combined to describe location-specific PA/ST; iii) the data and variables that can be extracted; and iv) the ability of the data to probe patterns of PA/ST in the indoor environment, by presenting preliminary findings regarding location and PA/ST from two groups of office workers in two UK based office buildings.

## Methods

This study is part of the Active Buildings project[21] which aims to examine associations between the design of the indoor environment, specifically office environments, and PA/ST. Detailed information of the overall study protocol is available in the Active Buildings protocol paper [22]. Participants in the work described in this paper are drawn from the sub-sample who agreed to take part in the objective monitoring arm of the Active Buildings project.

### Ethics statement

Ethical approval for this study was obtained from the UCL Research Ethics Committee (4400/001) and written informed consent was provided by all participants.

### Technologies

This study utilised the OpenBeacon active RFID system [23] for indoor tracking and the ActivPAL system [24] for accelerometer information. Here we give the requisite technical overviews of both technologies.

### ActivPAL overview

In this study we measured PA/ST using the ActivPAL accelerometer/inclinometer, which can characterise sitting, standing and stepping time as well as the number of steps and sitting to standing transitions. The ActivPAL is a widely used and validated tool [25–32] for measuring PA/ST. It has been utilised previously to investigate PA/ST in office workers [33] and in studies of free living adults [34]. The sensor itself is a small rectangular device worn continuously on the thigh including during bathing and sleeping. In this study participants were instructed to wear the ActivPAL device continuously for the duration of the monitoring period. On completion of the monitoring wear protocol, ActivPal data were downloaded at the research centre. The ActivPal records movement data at 20 Hz and can deliver PA/ST data in several formats. Movement data were opened in the ActivPal interface program and exported in the ‘events file’ format that lists time stamped records of each step taken and each transition between any state of sitting, standing and stepping. Such data has a time resolution of one second. All data collected were visually inspected for unusual episodes, none were observed. Compliance in wearing the device was confirmed by self-report.

### Open Beacon Overview

**Fig 1:**
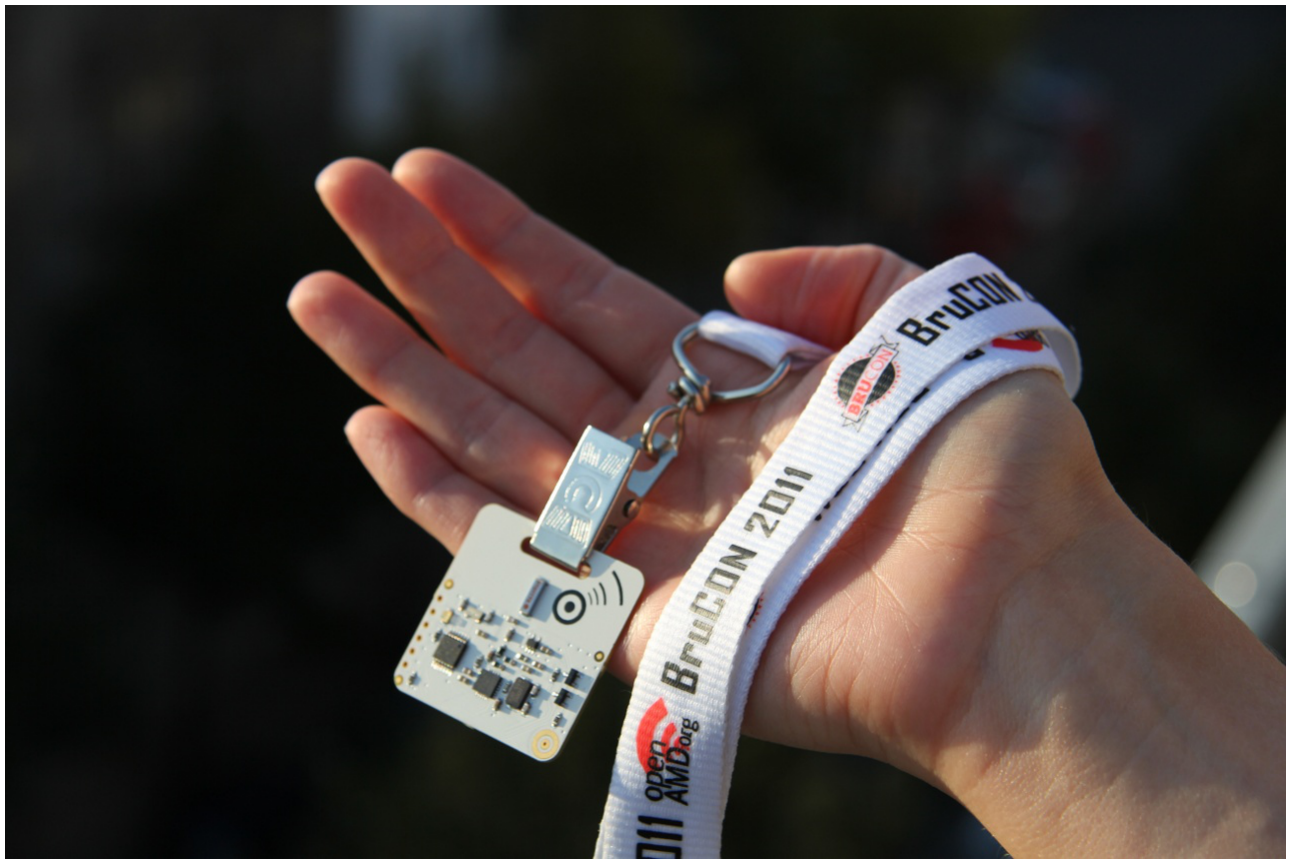
Open Beacon tag. A tag used in the Open Beacon system.

The Open Beacon system is a RFID platform that identifies proximity through interactions between pairs of lightweight, unobtrusive ‘tags’ (shown in Fig 1), detected by ethernet-connected readers distributed within the study buildings. The readers transmit the data to the research centre. This system has been widely used within the context of the Sociopatterns project [35] to detect face-to-face human interactions [36–42]. The system was chosen in this study for both cost considerations and the flexibility that the multi-tag setup permits, such as the possibility to simultaneously investigate social contact between individuals alongside location.

In this study some of the tags were worn by tracked participants on a lanyard around the neck (denoted participant tags), while others were affixed throughout the environment (denoted stationary tags). Participants were instructed to wear the participant tags continuously during waking hours in the monitoring period. All tags possess a unique identifier and frequently broadcast transmissions, denoted type A transmissions, containing their unique identifier into the surrounding area. The participant tags also frequently listen for type A transmissions from other tags, but only during defined time windows and cannot transmit and listen simultaneously. The power of the type A transmissions is tuned so that a listening participant tag can only receive them if the tags are in close proximity (~1-2m). When a tag receives a type A transmission, the tag identifiers are relayed for storage by means of a secondary mechanism and additional infrastructure. When a participant tag receives a type A transmission from another tag it first creates a record of that interaction that includes the unique identifiers of both the receiving and the transmitting tag. Such records are then included in an additional and distinct type B transmission broadcast only by the participant tags, which also contains the participant tag’s identifier. Type B transmissions are frequently broadcast into the surrounding area, regardless of whether a type A transmission has been received, and are received by separately installed base stations called ‘readers’. The readers are installed in the tracking environment as part of the wider infrastructure of the system. The readers rely on access to an existing local area network (LAN) infrastructure in the area being tracked. Using the LAN infrastructure, records of both type A and type B transmissions are sent from the readers to a central computer which stores and time stamps them with a precision of one second. Type B transmissions have a much larger effective range (about 15m). Consequently, assuming perfect communication of both transmissions, the central computer receives and stores reports of type B transmissions from participant tags that are within approximately 15m of a reader alongside reports of type A transmissions from participant tags that are within approximately 1-2m of a stationary tag. Spatial information can then be inferred from these reports since they indicate proximity between participant tag and the stationary tags and readers. However, the ability to do so depends on the locations of the stationary tags and the readers as well as the consistency and reliability of the transmission reports. Consequently to specify fully the implementation of the system both a deployment strategy for the tags and readers alongside an inference strategy for location from the reports they provide are required.

### Deployment strategy of the tracking system

The deployment strategy for the readers was to ensure that that any point in the area to be tracked was within 15m of at least one reader and to ensure that the distance between two adjacent readers was no more than 15m. The intention of such a strategy was to ensure that participant tags would always be able to successfully deliver type B transmissions to at least one reader, with as much redundancy as was feasible given limited resources, within the entire office area where participants were being tracked. We denote this area the ‘wider tracking area’. In this study the wider tracking areas comprised all departmental desk areas and facilities where the participants worked including printers, toilets, kitchens and informal meeting areas. However, it did not cover the building more widely such as the lobby and entrance.

Finer accounts of location can be inferred if type A transmissions are received. To do so reliably therefore requires a stationary tag to be close to the participant at any given time. Consequently the deployment strategy of stationary tags was to install them such that, ideally, there was a stationary tag every 1-2m throughout the wider tracking area. The nature of the tag technology means that the reliability of type A transmissions is strongly dependent on contextual details such as surrounding morphology/materials and tag orientation. Therefore the tags were installed in as many distinct orientations as possible to maximise the possibility of successful type A transmissions. Whenever a tag or reader was installed its position was marked on a floor plan of the building, and assigned an (x,y) location.

### Location inference strategy

Following a given deployment of readers and tags, the reports of type A and type B transmissions can be used to identify the location of the participant tags algorithmically. In this study this was achieved with a sequence of Matlab scripts, which, for each participant, read in time stamped lists of type A and type B transmissions, read in the ActivPAL data, combined the data into a single structure and performed the inference strategy. Raw transmissions from the tracking system were natively stored in pcap files and then converted into JSON data objects by means of open source software called the OpenBeacon Tracker API [25]. Lists of type A and type B transmissions for individual participants were obtained by accessing the data objects through the MongoDB database software and Python scripting language. The novel procedures used in this study for the inference strategy are described below. Detailed descriptions of all such steps are included in section A in S1 Appendix.

### Alignment of tracking and accelerometer data

A crucial and, to our knowledge, novel step in our location inference strategy is to utilise not only the type A and type B transmissions data, but also information from the ActivPAL accelerometer/inclinometer. The ActivPAL data provides a time-stamped sequence of activity codes whilst the Open Beacon provides a time-stamped sequence of type A and type B transmission reports. Before any inference of location is performed it is necessary that we first require the time-stamps for the activity codes to refer to the same real world time as the tracking system’s transmission reports. Each ActivPAL is responsible for its own time keeping and so a correction must be applied to each participant’s data. We allow, check and correct for constant and linear discrepancies in time keeping between the ActivPALs and the tracking system. This is to allow for any isolated, non-persistent, sources of misalignment through the constant term and a potentially persistent inaccuracy through the linear term. These corrections are determined, individually for each participant, through visual inspection within the Matlab environment and implemented by a time correction term, defined individually for each participant, in the Matlab scripts that perform the inference strategy. They are confirmed by demanding mutual consistency, across the whole data stream, between ActivPAL codes and type A reports that unambiguously indicate large-scale changes in positions that could not arise due to noise or small errors. Typically the linear correction term corrected a discrepancy of around 2 seconds per day.

### Inference from type B transmissions: presence in wider tracking area

Owing to the longer range of the type B transmissions, and fact that the participants were instructed to wear their tracking tags at all times both in and out of the office[22], the type B transmissions have been used to determine when the participants had entered and left the wider tracking area. As such the use the type B transmissions in this way allowed pragmatic identification of time spent exclusively in office areas.

### Inference from type A transmissions: location within the office

**Fig 2:**
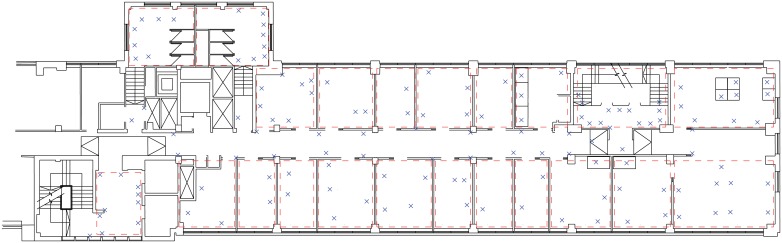
Deployment of stationary tags and immediate tracking areas. A floor plan of a participating organisation. Black solid lines indicate walls and office structures, blue crosses indicate stationary tags and dashed red lines indicate groupings of stationary tags into non-overlapping regions denoted ‘immediate tracking areas’ with which participants can be associated.

The reliability of type A transmissions is strongly dependent on contextual details. Whilst the range of type A transmissions is typically around 1-2m, this bound is not absolute, often being smaller and occasionally larger. The range is affected by factors such as the relative angle of the tags, the presence of blocking or reflecting bodies/structures and a certain inherent variability in the tag behaviour. For example, the human body blocks type A transmissions meaning that participant tags worn on the participant’s chest cannot receive transmissions from stationary tags behind them. In addition, because tag transmission and listening functions are intermittent, even when in effective range, a successful transmission is not guaranteed, but increases in likelihood with continuous proximity. As such it is possible to have type A reports that are from stationary tags that are not closest to the participant or an absence of type A reports altogether. This variable, but typically short range, behaviour means that many tags are required for adequate coverage & reliability, and that inference of location based on single tag interactions may be unreliable. Therefore our strategy is to infer location from multiple tag interactions and achieve this, given a constant number of tags, by lowering the spatial resolution. This is implemented by defining non-overlapping regions of the wider tracking area, each of which contains multiple stationary tags, and inferring which of these regions the participant is in. Such regions are denoted ‘immediate tracking areas’ within the wider tracking area. The rationale for such an approach is that, by containing many stationary tags in different positions/orientations etc., inference of presence within an immediate tracking area can be inferred from single or multiple interactions and provides redundancy for noisy or absent tag interaction data. In this study the immediate tracking areas were chosen to correspond to spaces important to the research question. These are separate rooms defined by walls, or functional areas such as kitchens or banks of adjacent desks in open plan offices. An example is given in Fig 2. The use of this strategy in our study allows explicit definition of movement as changes between immediate tracking areas indicated by the tracking procedure.

Following establishment of the immediate tracking areas we can then infer location for the participant tags. A key step that we utilise in order to overcome periods of missing data and periods of contradictory or noisy data is to establish when the participants were stationary for continuous periods of time through examination of the ActivPAL data. We reason that during times in which the participant is known to be stationary, he/she should only ever be associated with one of the immediate tracking areas and that he/she should be associated with that immediate tracking area for the entirety of that time since an inactive participant should be incapable of changes in location. This allows us to treat the location during these times as a single inference by asking which single immediate tracking area is most supported by all of the type A transmissions during that time. For instance, this means that the location of a participant during long periods of sitting, but where very few type A transmissions were recorded, can be confidently asserted, for that entire time, based upon those few reports. Without such an approach it might only have been possible to identify the location for short instances with large intervals when there would be insufficient data to make an inference. Similarly a long period of sitting where the type A transmissions seem to indicate some changes in location between adjacent immediate tracking areas, can be identified as time spent in one location. Without such an approach some spurious changes in location may have been identified.

In the periods of time between such stationary behaviour, reports of type A transmissions are used to infer locations by performing a moving average of the positions associated with the relevant stationary tags within a defined time window alongside ActivPAL activity information to make the most plausible inferences. Details of the entire inference strategy are described in section A in S1 Appendix.

### Resultant data structure

**Fig 3:**
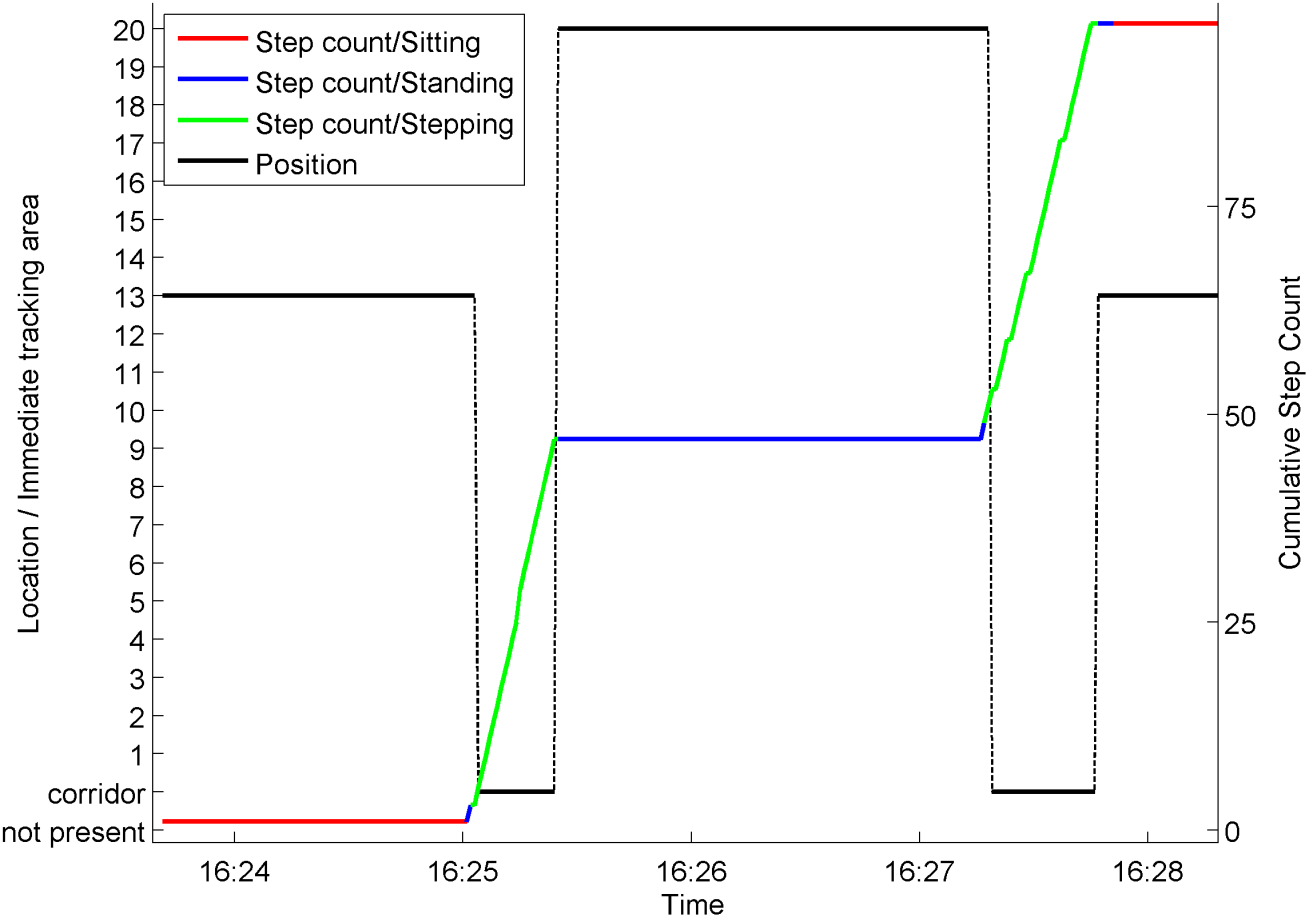
Final data structure capturing typical participant behaviour. A participant is sitting in immediate tracking area 13, stands and walks through a connecting space to immediate tracking area 20 and stands. Later they walk back to immediate tracking area 13 through connecting space and sit back down.

The resultant data structure for each participant indicates, for each second, an activity code from the ActivPAL, the cumulative number of steps taken and a location code. The location code indicates either an individual immediate tracking area, absence from the wider tracking area or presence within the wider tracking area, but not within any immediate tracking area. If the participant is within the wider tracking area but not in one of the designated immediate tracking areas, then they are deemed to be within connecting areas such as corridors. An illustration of this final data structure arising from the overall procedure for a typical individual example of movement for one participant is given in Fig 3.

It should be noted that localisation of the participant within connecting space can sometimes arise when tag interaction data are sparse or missing, perhaps due to a damaged or improperly worn participant tag. A heuristic for data sparseness is used as a final exclusion step to remove participants with data deemed unreliable or insufficient from the sample. Details can be found in section B in S1 Appendix.

### Study details

#### Participants and populations

This study consisted of two separate cases of buildings, each accommodating a distinct organisation and having a separate installation and usage of the tracking system, ActivPAL devices and inference strategy. In each case study ActivPAL devices and tracking tags were administered to 19 participants who were monitored for five working days within a 7 day period. The participant organisations were university departments. Participants were all desk-based with roles comprised of administrative and research staff. Each organisation allocated specific desks to their workers, which were normal sitting desks that were not designed for standing whilst working.

#### Direct Observations

In order to confirm preliminary evidence for accuracy a comparison between the results of the tracking methodology and direct observations is performed. When assessing the accuracy of the use of the Open Beacon system and its combination with ActivPAL data, it should be highlighted that the performance is dependent on specific contextual details as noted earlier. These can affect the deployment strategy, the ability to implement it, the performance of the tags and the reliability of the inference strategy. For instance, one cannot expect equivalent performance in localising participants within ‘rooms’ if what constitutes a room differs (in size/morphology etc.) in different applications of the system. Therefore it is not possible to ‘validate’ such a system ‘out of the box’ for all possible future usages in the sense usually associated with a new technology. However, we emphasise that this does not mean that one cannot assess the system’s performance in a specific context such as in a particular building, which we do in this paper without claiming validation in a broad sense.

As part of the deployment of the tracking system, trained observers recorded the time-stamped incidence of participants’ presence in six specifically monitored locations, each identifiable as an immediate tracking area. These periods of presence we call ‘trips’ and can be identified using the resultant data from the tracking methodology. The areas corresponded to WCs and kitchens within the case study buildings. A single trained observer remained within each location during documented times and noted the times participants entered and exited the location identifying participants by visible ID numbers worn by each of the participants. All study participants were instructed to follow the Active Buildings protocol [22].

To assess the performance we provide the total number of observed trips according to the tracking methodology and the total number of trips according to direct observations during the documented times when presence was noted by the observers. We also provide the number of specific trips noted in direct observations, but absent from the tracking results and the number of specific trips that are detected by tracking methodology, but absent in direct observations. From these quantities we estimate and report the probability of a false positive, the probability that a given ‘trip’ in the tracking results is not found in direct observations, and the probability of a false negative, the probability that a direct observation is not found in the tracking results.

#### Statistical Analyses

Using the tracking methodology, sequences of coincident location and PA/ST data are generated for each participant. These sequences are used to derive different variables. These variables can be the number of specifically identified patterns in location, a quantity attributable to some identifiable pattern in location or averages of PA/ST over that sequence amongst others. In this paper we treat all such variables in two distinct ways. The first is to generate sample wide variables whereby such quantities are averages across the entire data set formed from all the sequences of location and PA/ST from each participant. The second arises whenever there is discussion of variation within our sample, by means of descriptive statistics and associations, wherein an instance of a variable is created for each participant derived from their sequence of location and PA/ST. In all such instances any averages stated are the averages of such participant variables and any n given is the number of participants. Finally, where associations are investigated simple linear regression is performed upon appropriate pairs of participant variables and regression coefficients are reported as effect sizes with appropriate units.

## Results and Discussion

Of the 38 total participants, five did not meet the data final criteria examining data sparseness indicating poor accelerometry or tracking data and were therefore excluded leaving a working data set of 33 participants. Further details and individual participant breakdowns of the following results are given in section B and tables A, B and C in S1 Appendix.

### Comparison with direct observations

The direct observations noted by the observers are contrasted with records of presence derived from the resultant location data in Table 1. Full participant results are given in table A in S1 Appendix. Note is made of the number of agreed upon events—entries in the direct observations that match reports from the tracking system, the number of observations that were not identified in the tracking system reports and the number of tracking system reports that were not identified in the observations. We observe an agreement with direct observations approaching 90% with the probability of both false negatives and positives being around 13%, a level of agreement we deem appropriate for this work. However, we emphasise that the variable being tested here is derived from the final data structure containing location and activity rather than a comparison of its raw format.

**Table 1:** Agreement with direct observations.

	Building 1 Kitchen	Building 1 WC	Building 2 WC 1	Building 2 Kitchen 1	Building 2 WC 2	Building 2 Kitchen 2	Total
Direct observation count	93	22	9	29	23	83	259
Tracking report count	88	19	10	36	20	85	258
Number of direct observations that do not appear in the tracking data	16	3	0	1	4	12	36
Number of tracking reports that do not appear in the direct observations	11	0	1	8	1	13	35
Number of tracking reports and direct observations in agreement	77	19	9	28	19	71	223
Fraction of direct observations matched to a tracking report.	0.828	0.864	1.000	0.966	0.826	0.855	0.861
Fraction of tracking reports matched to a direct observation.	0.875	1.000	0.900	0.778	0.950	0.835	0.864
Estimate of the probability of false positives	0.125	0.000	0.100	0.222	0.050	0.165	0.136
Estimate of the probability of false negatives	0.172	0.136	0.000	0.000	0.174	0.145	0.139

Agreement with direct observations measured at six locations within the two buildings in this study. Estimates of false positives and negatives are determined by the fraction of tracking reports that are absent from the direct observations and by the fraction of direct observations that are absent from the tracking reports respectively.

### Illustration of novel results

#### Overview

Here we demonstrate how the resultant data can be used to build pictures of location and movement, derive precise measures of PA/ST within defined locations and derive complex variables that characterise the usage of different locations within a tracked building.

**Fig 4:**
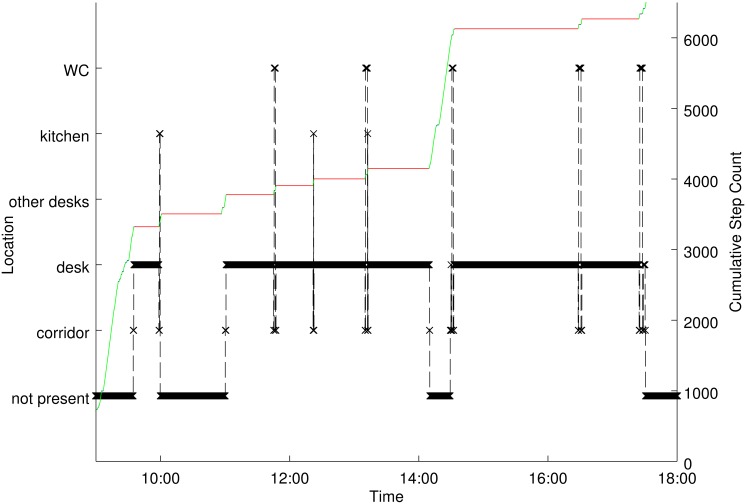
Typical working day behaviour of a participant. Location information against time for one working day for a single participant. Also shown is the cumulative step count on the right hand y-axis with the colour indicating activity information: red indicates sitting, blue standing and green stepping.

As described, the resultant data is a time series of location and PA/ST. Before any further variables are derived one can build illustrations of activity that illustrate the behaviour one might expect within office environments. For example, the combined location and PA & ST information for an individual participant for an entire working day is shown in Fig 4.

The data generally show long periods of sedentary behaviour at a desk location interrupted by short duration trips to other locations that incur both stepping and standing. This behaviour was typical for all participants although the locations visited and the number of trips to such locations showed significant variation.

In Fig 4 we can see that, between 14:00 and 15:00, a large number of steps were taken in a short time beyond the wider tracking area. Such behaviour may, for instance, reflect a lunch break. If one wished to characterise accurately the levels of PA/ST exclusively within office environments it would be important to exclude such a period of time. Without coincident location and PA/ST data this is challenging, as it would require accurate self-reporting of entry and exit times. Even when assuming complete adherence to self reporting, even very small quantitative inaccuracies in timing reports could significantly alter estimates of PA/ST owing to the radically different profiles of PA/ST in the two time periods as illustrated in Fig 4. The use of the tracking system and alignment with the ActivPAL device allows accurate detection of entry and exit times and therefore construction of PA/ST variables formed exclusively from data when the participant was in the wider tracking area ie within the office environment. Similarly this approach can be used to construct equivalent variables for individual locations, identified as immediate tracking areas or sets of them, allowing creation of accurate activity profiles of different locations within the office environment.

Further variables of interest can be derived from the time series data of location and PA/ST. We have already examined one such derived variable, the number of trips to a certain location. This is derived from the data by searching for contiguous periods of time where the participant was associated with such a location. However, since all such movements/activities are time stamped and can be viewed within the context of the location information that precedes and follows it, many nuanced variables can be produced. This includes, but is not limited to, the time spent in certain locations, the time when trips to locations occur, the statistics of the time between trips to certain locations and patterns in the trip sequences exhibited by individuals. We illustrate some of these possibilities in the next section.

#### Illustrations of derived tracking variables

Here we discuss some of the derived variables that can be produced with such data. The general patterns of movement that are observed are well illustrated in Fig 4. Much of the time is spent sitting in desk areas broken up with short trips to other locations. This suggests variables concerning trips to different locations may be most useful in characterising movement in these environments. As such we consider derived variables related to such trips and for illustrative purposes consider two separate locations: WCs and kitchens, locations whose usages might be expected to differ because of the expectation that WC trips might be largely driven by physiological factors whilst kitchen trips may be driven by the desire for socialisation and other voluntary factors. Figures referred to in this section can be found in S1 Appendix.

First, we examine the timing of such trips by looking at the statistics of when such trips occur for participants in each case study building. Such statistics are given in Fig. A in S1 Appendix. Next we construct a variable concerning the length of time spent in each location. The statistics over all participants in the case studies are shown in Fig. B in S1 Appendix.

Finally we present a more nuanced derived variable pertaining to the time between trips to each location. Here we examine the statistics of the time between trips to each type of location, but also examine such a statistic for the first (defined as the time since entry to the wider tracking area), second, third and fourth such trip of the day. The motivation being that if the statistics for all such trips are identical then the usage of such a location might be considered to be broadly uniform throughout the day. However, if it is not then this might indicate an adaption of behaviour throughout the day, perhaps revealing aspects of routine that are exhibited in the participants. Such data are presented, for all participants in both case study buildings in Fig. C in S1 Appendix.

In broad terms we do see differences in the utilisation of such locations within our sample and demonstrate that the tracking system and inference strategy is able to identify such differences. For instance our results would suggest that, on average, trips to kitchens are rather fleeting and there is no typical time spent there in contrast to trips to WCs where there is a typical time spent in such locations. Similarly the statistics of the time spent waiting between kitchen trips seems to be broadly uniform throughout the day whereas the time between WC trips can be seen, on average, to increase throughout the day. Interestingly, the first trip of the day to both locations occurs much sooner than subsequent trips perhaps reflecting a tendency to visit such locations when arriving at the office as part of a daily routine.

#### Preliminary location-specific findings and associations between PA/ST and movement

Here we quantitatively investigate both the PA/ST behaviours that are seen across the wider tracking area in our case studies, but also the PA/ST behaviours seen in particular immediate tracking areas and derived location variables in the form of trips to various locations. With such data we intend to make preliminarily characterisations of the patterns of PA/ST that might be expected within office environments, albeit based on a small sample.

First, we assess the sample-wide descriptive statistics of the most important PA/ST and derived trip variables for the whole sample from the two case study buildings (n = 33). Full participant results are included in tables B and C in S1 Appendix. Such results characterise time spent within the wider tracking area and are illustrated in Table 2.

**Table 2:** PA/ST and movement within the wider tracking area.

	Sitting time (minutes per hour)	Standing time (minutes per hour)	Stepping time (minutes per hour)	Steps per hour	Sit to stand transitions per hour	Kitchen trips per hour	WC trips per hour	Other desk trips per hour	Trips from desk per hour
Mean (standard deviation)	46.2 (10.7)	11.4 (10.9)	2.4 (1.0)	200.9 (82.9)	3.1 (1.5)	0.96 (0.51)	0.38 (0.20)	0.96 (0.66)	1.60 (0.63)
Median (range)	49.2 (5.6–56.5)	8.4 (2.4–49.7)	2.2 (0.9–5.9)	177.8 (69.5–466.5)	2.7 (1.0–8.1)	0.83 (0.27–2.74)	0.39 (0.0–0.79)	0.83 (0.0–2.55)	1.50 (0.50–4.07)

Average hourly duration of PA/ST, number of transitions and trips, derived from time within wider tracking area for all participants in two buildings (n = 33).

**Fig 5:**
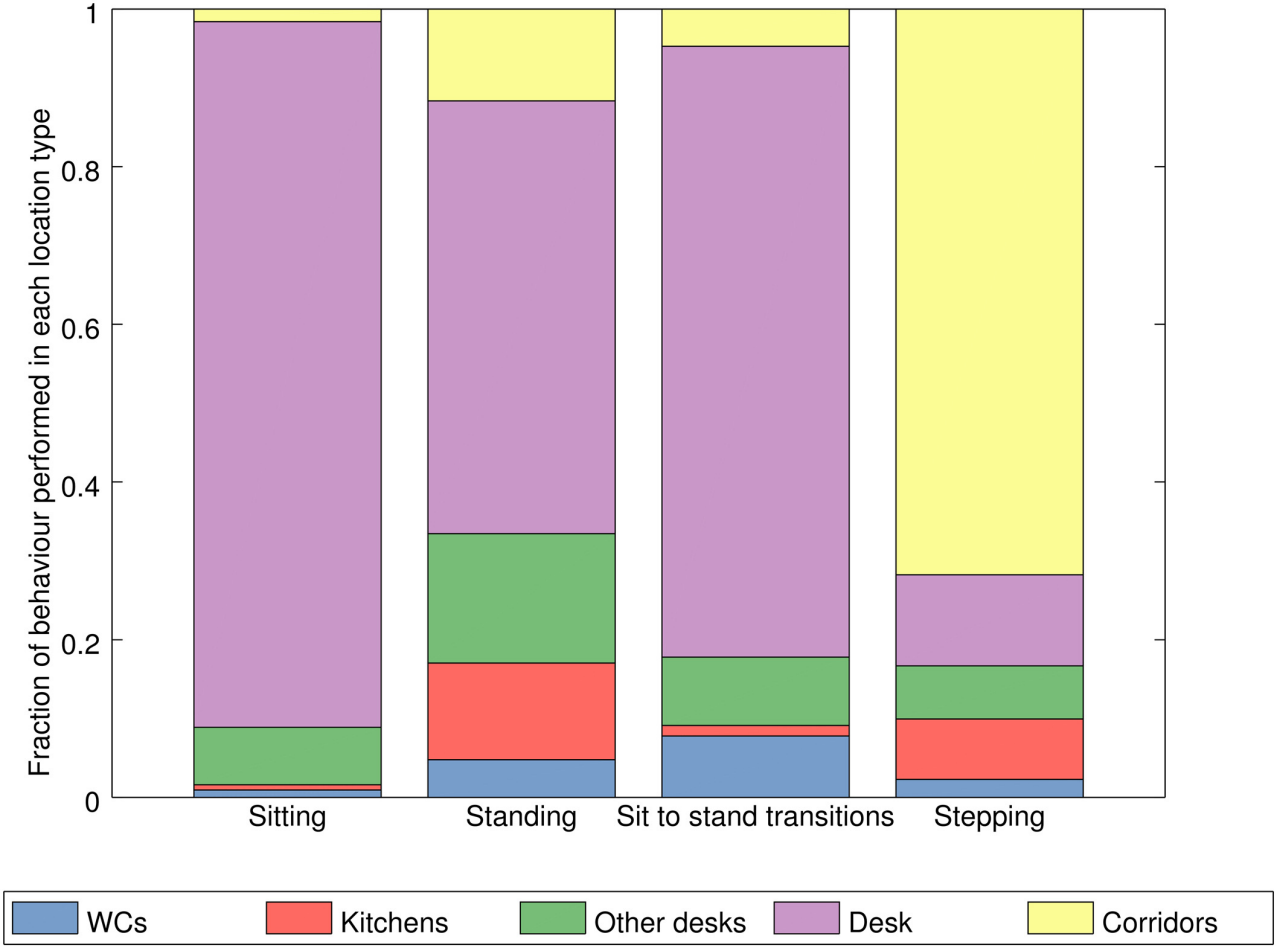
Activity behaviour at different locations. Distribution of distinct PA/ST behaviours across categories of location in the wider tracking area (n = 33). Each behaviour (sitting, standing, stepping, sit to stand transitions) is to be considered separately.

Building upon such descriptive statistics we can ask how the measures of PA/ST are distributed amongst various types of location commonly found in office environments. A location based distribution of such measures over all participants in both case studies are shown in Fig 5.

**Fig 6:**
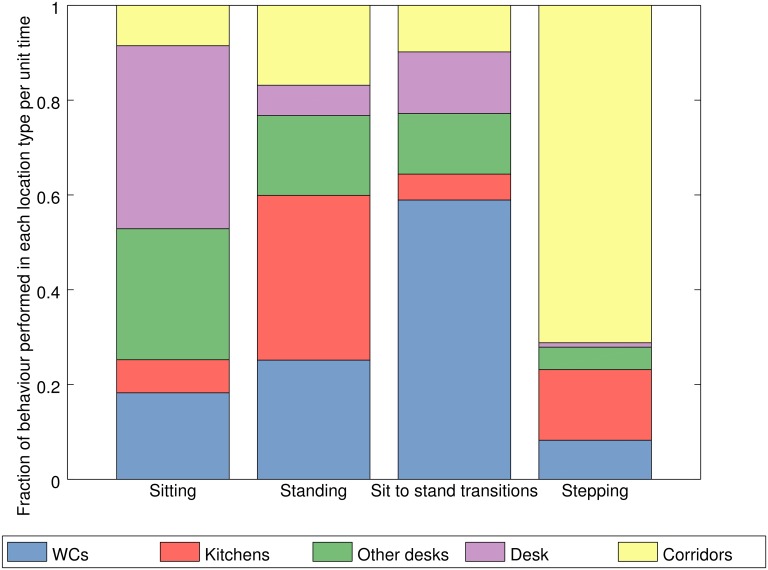
Time reweighted activity behaviour at different locations. Distributions of PA/ST behaviours across categories of location reweighted to counter the effect of unequal amounts of time spent in each location (n = 33).

For example, around 86% of all sitting time within the wider tracking area occurs in desk areas and 12% of all standing time occurs in kitchen areas. As one might expect, desk areas are locations where the majority of sitting occurs while undetermined locations, taken as a proxy for corridors and connecting spaces, are most associated with stepping. Perhaps surprisingly, the desk areas are also where the majority of standing behaviour occurs. However, it should be highlighted that whilst in absolute terms participants spend the majority of their total standing time at their desks, this is not necessarily because they stand at their desks proportionately more than they stand elsewhere, but more likely due to the large amount of time that they spend at their desk overall. For the participants in our sample, on average, 76.7% of time within the wider tracking area (taken as a proxy for office time) was spent at their desk area, 8.7% in connecting areas, 9.7% at other desk areas, 3.2% in kitchens and 1.7% in WCs. As such, we can scale the PA/ST outcomes by the time spent at each type of location to produce a normalised value for each activity at each destination. To do so we consider the fraction of each PA/ST measure performed at each location as if equal time were spent in all of them. Such results are presented in Fig 6.

For example, given an equal amount of time spent in both the participant’s own desk area and other desk areas, we would expect, on average based on our sample, around a third more sitting and around half as much standing in the participant’s desk area compared to other desk areas. The findings also suggest that whilst connecting spaces still dominate in terms of steps taken, kitchen areas can be seen to outstrip others in standing time on a per unit time basis.

Finally we use such a description of the location dependent nature of office PA/ST as motivation for investigating associations between movement variables such as trips to certain locations and PA/ST variables such as the number of steps taken per hour. For instance, both Fig 4 and the dominance of corridors and connecting spaces in terms of the number of steps performed shown in Figs 5 and 6 might suggest that trips to destinations dominate how steps are accumulated in such environments. We can then begin to investigate which such locations are most associated in this regard. Similarly we see that standing time at kitchens is, in relative terms, higher than in other locations. As such we can ask whether we observe a meaningful increase in standing time in those who visit the kitchen more.

Owing to the small sample size and exploratory nature of the study only simple univariate regression models are utilised. This is sufficient for illustrating the simple associations we are investigating in our dataset, but we note the limitations, notably the absence of any corrections for potential confounders should one wish to investigate such relationships more completely.

Several strong associations in our exploratory sample can be identified. The number of trips per hour to various locations was seen to correlate with the number of steps performed per hour. The results for such trips are shown in Table 3.

**Table 3:** Associations between step counts and trips to destinations.

Trip type	Effect size (steps per hour per trip per hour)	R value	Significance (P-value)	95% CI*
To Kitchen	103.2	0.646	<0.001	[59.33, 147.05]
To WC	55.1	0.133	0.453	[-92.57, 202.86]
To Other desk	85.9	0.672	<0.001	[50.13, 116.29]
Away from desk	89.3	0.691	<0.001	[55.69, 122.86]

Associations between steps per hour within the wider tracking area and trips to/from types of location. In all cases the dependent variable was the average number of steps per hour performed in the wider tracking area by the participant. The units of the effect size are steps per hour increase for unit trips per hour increase. In all cases n = 33.

*95% confidence interval.

Trips away from desks generally are associated with large and significant increases in the number of steps performed. This trend is reflected in trips to the specific location types of kitchens and other desk areas (e.g. colleagues), however this trend is not observed in trips to WCs where there in no significant association. Trips appear to be associated with step counts and the difference in association strengths may suggest something of the behaviour of participants based on the type of trip being performed. Perhaps the difference reflects differences in the movement behaviour associated with voluntary trips (to kitchen/colleagues) and imperative trips (to WCs).

Finally we examine whether trips to kitchen areas, identified as areas with strong standing behaviour, can be seen to influence individual standing metrics. We note that standing behaviour appears to be subject to large amounts of individual variation, perhaps determined by individual behaviours related to habitual standing in desk areas. As such we provide two such analyses with and without strong standing outliers removed. The associations are shown in Table 4.

**Table 4:** Associations between standing and trips to kitchens.

Participant exclusion	Effect size (minutes of standing per hour per kitchen trips per hour)	R value	Significance	95% CI*
n/a (n = 33)	8.4	0.43	0.011	[2.04, 14.76]
Participants with >12 min p/h standing time excluded (n = 23)	3.36	0.579	0.004	[1.20, 5.58]

Associations between trips to kitchen areas per hour and minutes spent standing in the wider tracking area per hour. Effect size is minutes per hour increase in standing per unit increase in trips to kitchen areas per hour. Exclusion field describes the condition for removing outliers.

*95% confidence interval.

There appears to be a strong relationship with kitchen usage and standing behaviour, but note that the association becomes stronger, albeit with a smaller effect size, when considering those who typically do not show strong standing behaviour. This may be plausible since any standing that occurs in kitchens will be a larger proportion of standing time in those who stand less overall.

## Discussion

Following the presentation of our data and results we now discuss wider aspects of this research approach including its performance, practicality, reliability and the relation to the research questions that one might wish to investigate. To the best of our knowledge, the approach we have implemented is novel and we have presented data that would be challenging or infeasible to source by other means. For instance, an accurate assessment of step count based on where they were performed, requiring each individual step to be classified according to where it was performed, would be challenging to obtain even with well trained observers. Particularly, we believe that being able to provide objectively measured data—as opposed to self-reports—regarding changes in location (e.g. trips to specific destinations), and entry and exit times to the immediate office area could be of great utility to researchers. Further we have illustrated how the combination of time-stamped location data with information on sitting, standing and stepping in order to characterise, in fine detail, where and perhaps to infer how and why PA/ST is generated, could potentially be an asset in understanding the determinants of PA/ST in indoor environments. In particular we have presented data, which, for our small sample in this exploratory study, strongly suggests that trips to certain destinations are a key mechanism in the generation of PA/breakup of ST in office environments. Whilst this may be an expected result it provides a clear focus for further investigations and lends credibility to the validity of such a research technique. Applications of such a technique might include the investigation and evaluation of whether and how specific design features or interventions to promote physical activity and/or sedentary behaviour reduction influence the mechanisms that generate and spatial distribution of PA/ST.

Practical and technical considerations associated with the approaches illustrated in this paper must be highlighted. A notable issue is the performance of the tracking system and inference procedure (e.g. in terms of accuracy and resolution), and the related difficulties discussed earlier in establishing a ‘validation’ protocol such that one could reasonably expect consistent performance in future usages in different environments, without the need for extensive ad-hoc calibration and testing. This is an inherent feature of a system where installation/inference details must be determined at the deployment stage. However, some aspects are likely to occur with many similar systems to some degree, such as the possible variation in performance arising from differences in building morphology & materials, the ability to install infrastructure and contextual location definitions.

Finally we highlight the technical and practical issues associated with using the indoor tracking system in combination with accelerometers and how they relate to the accuracy that can be achieved. A key practical step required to combine of the technologies was the alignment of the data from both the tracking system and the ActivPAL device. Such a procedure is a highly time consuming task, but an unavoidable consequence of requiring both activity and location data from two separate devices. The specific tracking system we utilised has features that present some practical and technical challenges. For instance stationary tags need to be installed throughout the entire space and at regular intervals along with readers. This too can be time consuming and is vulnerable to environmental and institutional limitations on the ability to install the equipment, as well as to some mild forms of intentional or accidental vandalism. Such issues may sometimes be exacerbated by concerns about invasive monitoring amongst participants and non-participant residents within the participant buildings, which may often be difficult to alleviate entirely. Technological challenges also concern the distributed nature of the system coupled with location inference being proximity based. The system itself does not report locations or positions, but tag interactions in a raw format. Whilst this provides a considerable amount of flexibility, the researchers need to devise their own inference strategy from such interactions, dependent on how the tags were installed, which can be both challenging and time consuming. Such reliance on distributed tag interactions also has consequences on performance. This is largely because the data from which location is inferred is not delivered continuously, but only exists when type A transmissions are received. Limited tag resources, limited ability to install them and contextual tag performance means continuous type A transmissions cannot be guaranteed. This means that there can be periods of time, of varying length, for which there are no location data. Such a feature can lead to ambiguities. For instance a period of missing data may arise from a participant turning away from the nearest stationary tag, or it may arise from distinct movement that did not generate any tag interactions. Based on such experiences we would therefore recommend that an effective tracking solution ought to be able, reliably and continuously, to deliver relevant data on a short timescale compared to the movement of the participants, such as every second, and that its ability to do so should not depend on the location or behaviour of the participant. We also recommend that, ideally, such a solution should perform noise reduction and location inference natively. This would mean that the delivered data is in the form of a location or position not only removing the burden of such a step from the researcher, but allowing for standardisation of such a process throughout research studies and in the literature.

## Conclusion

In this paper we have introduced a research approach for assessing aspects of location-based activity/sedentary behaviours of participants within office buildings. This approach consisted of using an indoor tracking system in conjunction with the ActivPAL device to provide a time-stamped sequence of location-based physical activity and sedentary behaviour outcomes within offices. The use of such a system and associated procedure has been compared to direct observations and we have shown how this information can be utilised to build a data set that can be used to investigate detailed questions about PA/ST within the workplace. Ultimately, one could examine whether location-specific PA/ST outcomes are independently associated with specific characteristics of those locations and/or of the wider physical environment. The specific system utilised here had a series of technical limitations—some of which are likely to occur in other systems using the same technology. We would not recommend the use of an analogous proximity-based location system for similar applications, unless a relatively low spatial resolution is acceptable and capturing participant interactions alongside location information is considered valuable for the research question. However, we have shown that the technology is now available to capture location information inside buildings used by office workers and that this can be combined with activity data to create variables previously unavailable for research.

## Supporting Information

S1 Appendix Additional Material. Appendix containing further information on the inference procedure, full participant results and supplementary figures. **Table A**: Summary of excluded data and the effect on utilised direct observations. **Table B:** Physical activity and sitting time data for valid data for included participants. **Table C:** Trip data using valid data for included participants. **Fig A:** Trip timing within the working day. Distribution of the time at which trips to kitchens and WCs occur for all participants within each building case study (n = 33). **Fig B:** Time spent at trip locations. Distribution of time spent in WCs and kitchens derived from all participant data across both case study buildings (n = 33). **Fig C:** Waiting time between trips. Distribution of time between trips to WCs and kitchens and between specific trip numbers derived from all participant data (n = 33).
(PDF)
