# Supplementary material for: Indoor Tracking to Understand Physical Activity and Sedentary Behaviour: Exploratory Study in UK Office Buildings
Source: PLoS One. 2015 May 20;10(5):e0127688. doi: 10.1371/journal.pone.0127688 (PMC4684195; doi:10.1371/journal.pone.0127688)
Supplement: S1 Appendix — Appendix containing further information on the inference procedure, full participant results and supplementary figures. Table A: Summary of excluded data and the effect on utilised direct observations. Table B: Physical activity and sitting time data for valid data for included participants. Table C: Trip data using valid data for included participants. Fig A: Trip timing within the working day. Distribution of the time at which trips to kitchens and WCs occur for all participants within each building case study (n = 33). Fig B: Time spent at trip locations. Distribution of time spent in WCs and kitchens derived from all participant data across both case study buildings (n = 33). Fig C: Waiting time between trips. Distribution of time between trips to WCs and kitchens and between specific trip numbers derived from all participant data (n = 33). (PDF) [file pone.0127688.s001.pdf]

Appendix S1

A Procedure

Here we describe the total inference procure following alignment of the type A and B reports and the ActivPAL data. Such a procedure is repeated for every participant.

Step one (association within the wider tracking area)

- Search through the aligned type B report data for times when reports are absent for a time greater than a minimum cutoff of 10 seconds
- Associate such times as being outside of the wider tracking area.

Step two (association during stationary periods)

- Within times where the participant has not been determined to be outside of the wider tracking area search through the aligned activPAL data for periods of time that are comprised entirely of standing or sitting and are at least 30 seconds long
- For each time period perform an average of the x and y positions of the stationary tags associated with each report in the time period. For instance if there are two reports from tag 1 with position $(10, 20)$ and one report from tag 2 with position $(15, 25)$ the average x position is $\bar{x} = (10 + 10 + 15)/3$ and the average y position $\bar{y} = (20 + 20 + 25)/3$.
- Find the stationary tag closest to $(\bar{x}, \bar{y})$ and then the immediate tracking area within which that tag is located.
- Determine the location of the participant for every second within such a stationary period to be that immediate tracking area.
- If no type A reports are within the period associate the participant with connecting space.

This procedure results in periods of time where the participant's location has been determined and interleaving periods where the participant is found within the wider tracking area, but their location has yet to be determined. In those interleaving periods the third step is followed.

Step three (association during remaining periods)

- For each second in the remaining interleaving time periods perform an average of the x and y positions of the stationary tags associated with

type A reports that occur within two seconds forwards and backwards in time from the time being considered (five seconds total). Again if there are two reports from tag 1 with position $(10, 20)$ and one report from tag 2 with position $(15, 25)$ the average x position is $\bar{x} = (10 + 10 + 15)/3$ and the average y position $\bar{y} = (20 + 20 + 25)/3$.

- Find the stationary tag closest to $(\bar{x}, \bar{y})$ and then the immediate tracking area within which that tag is located.
- Determine the location of the participant for the second being considered to be that immediate tracking area.
- If no type A reports are found, but no steps have been performed since the participant's last successful association with an immediate tracking area, associate the second with the last known immediate tracking area.
- If no type A reports are found and steps have been performed since the last known immediate tracking area, associate the time with connecting space.

Step four (noise reduction)

- Search through the entire data structure of ActivPAL codes and determined location.
- If the participant is associated with a location for less than five seconds replace the associated location of such times with connecting space.
- If the participant is associated with an unbroken sequence of different immediate tracking areas (i.e. with no time associated with connecting space) then the stationary tag positions within those immediate tracking areas are considered. If it is possible to choose a stationary tag from each immediate tracking area in the sequence such that all are within 2m of each other and fewer than five steps have been taken during that time the sequence is simplified. When this occurs the participant is associated with the most common immediate tracking area in the sequence for the duration of the sequence.

B Detailed Results

B.1 Data inclusion rules

To reach the final working data set two separate data streams had to be combined. However, each are separately vulnerable to missing data such that there are periods of time where one or more is missing or unreliable which can arise from failures to follow wearing protocol for either the tracking tag or the ActivPAL device or administering/collection of one device before the other. As such, to obtain the final data stream upon which we could perform our inference procedure, we procedurally ran through the combined data and removed sections where one or more types of data were missing or unreliable and did so in a specific sequence. First we established the total amount of time when there was tracking system data, identifiable by the first and last instances of presence

of the participant tag within the wider tracking area. We then removed periods of time where ActivPAL data was missing, typically arising from a subsequent administering of the device after the tracking tag. Next we removed periods of time for which the ActivPAL data was deemed to be untrustworthy usually from poor adherence to protocol such as taking the device off confirmed by participant feedback. Next we removed periods of time for which the tracking system data indicated poor adherence to protocol resulting in poor resultant data. For instance when a tag is found exclusively in one location despite plausible and significant activity according to the ActivPAL device it is possible the participant tag has been taken off. The inference procedure was performed on the remaining time consisting of reliable and coincident ActivPAL data and type A and type B transmission reports. From the resultant location data that resulted from the inference procedure, the proportion of the time within the wider tracking area for which the participant had been associated with an immediate tracking area was calculated. If this value was less than 80% the participant was excluded from the data set on the grounds of insufficient data quality. Each of these steps is illustrated for all participants in the two deployments included in this study in Table A. Also included are the effects such data exclusion had on direct observations for each participant.

building	particpant	tracking time (s)	coincident tracking & activPAL time (s)	time following removal of unusable data (s)	associated time	fraction associated	fractional inclusion criteria at 80%	direct observarions	consequent direct obervations ignored	working direct observations	tracking observations
1	1	54655	51201	51201	50194	0.98	Y	0	0	0	0
1	2	111974	102866	102866	91318	0.89	Y	7	0	7	8
1	3	125144	110677	110677	105322	0.95	Y	4	0	4	3
1	4	192687	181370	81520	76108	0.93	Y	17	0	17	15
1	5	140104	129899	129899	126995	0.98	Y	7	0	7	8
1	6	140829	132411	132411	129262	0.98	Y	4	0	4	6
1	7	633502	617085	27135	26304	0.97	Y	7	0	7	7
1	8	37082	25371	25371	24685	0.97	Y	4	0	4	4
1	9	63554	56361	56361	54674	0.97	Y	7	0	7	6
1	10	103629	94338	40965	38595	0.94	Y	6	6	0	0
1	11	85793	81949	81949	76101	0.93	Y	8	0	8	7
1	12	29708	29708	0	0	0	N	5	5	0	0
1	13	115836	103970	103970	100544	0.97	Y	2	0	2	2
1	14	138727	121045	111275	97129	0.87	Y	16	0	16	13
1	15	151303	140204	140204	137302	0.98	Y	6	0	6	4
1	16	107976	98570	98570	95103	0.96	Y	2	0	2	1
1	17	111750	106495	106495	95935	0.90	Y	15	0	15	16
1	18	91636	71812	71812	44277	0.62	N	0	0	0	0
1	19	87124	78179	78179	76587	0.98	Y	9	0	9	7
2	1	154245	154245	117603	110885	0.94	Y	18	7	11	15
2	2	97167	97167	97167	95075	0.98	Y	5	0	5	5
2	3	97441	97441	97441	81754	0.84	Y	7	0	7	8
2	4	62209	62209	62202	60132	0.97	Y	10	0	10	11
2	5	155943	155943	155943	150217	0.96	Y	5	0	5	5
2	6	0	0	0	0	0	N	0	0	0	0
2	7	26367	26367	26367	25128	0.95	Y	4	0	4	6
2	8	109307	109307	109307	104495	0.96	Y	13	0	13	10
2	9	44314	44314	44314	42003	0.95	Y	21	0	21	23
2	10	161180	161180	96153	89187	0.93	Y	14	0	14	16
2	11	151438	134113	89954	85470	0.95	Y	5	0	5	6
2	12	114159	114159	109649	107914	0.98	Y	6	0	6	6
2	13	285554	282700	194962	174935	0.90	Y	12	0	12	14
2	14	148655	148655	143040	140344	0.98	Y	8	0	8	9
2	15	32620	32620	29099	27976	0.96	Y	5	0	5	4
2	16	107207	107207	106895	101821	0.95	Y	14	0	14	10
2	17	175953	175953	47355	46926	0.99	Y	10	6	4	3
2	18	97728	70682	0	0	0	N	13	13	0	0
2	19	57498	52321	23904	0	0	N	7	7	0	0
Total		4601998	4360094	3103494	2890697	0.91		303	44	259	258

Table A: Summary of excluded data and the effect on utilised direct observations

B.2 Complete participant results

Tables B & C show, for all participants across both deployments, the measured movement variables such as time spent sitting/standing, the number of sitting to standing to transitions and number of steps per unit time. Also included are the number of trips to kitchens and bathrooms in absolute and per hour terms alongside sample wide mean and standard deviation figures where appropriate.

building	participant	total time in tracking area (s)	total time sitting in tracking area (s)	total time standing in tracking area (s)	total time stepping in tracking area (s)	total number of steps in tracking area	steps per hour	total number of sitting to standing transitions	sitting to standing transitions per hour
1	1	51201	46250	3513	1438	2244	157.78	27	1.90
1	2	102866	60124	36345	6397	9700	339.47	112	3.92
1	3	110677	77555	30044	3078	4572	148.71	148	4.81
1	4	81520	64717	13756	3047	4400	194.31	102	4.50
1	5	129899	109419	16590	3890	5274	146.16	172	4.77
1	6	132411	117934	10277	4200	6466	175.80	88	2.39
1	7	27135	23264	2965	906	1372	173.83	17	2.15
1	8	25371	19205	5252	914	1200	170.27	20	2.84
1	9	56361	47350	6905	2106	2814	179.74	63	4.02
1	10	40965	29708	9254	2003	2750	241.67	62	5.45
1	11	81949	64112	13545	4292	6110	268.41	122	5.36
1	13	103970	94884	5853	3233	4952	171.46	58	2.01
1	14	111275	96446	9400	5429	7188	232.55	62	2.01
1	15	140204	132159	5709	2336	3340	85.76	67	1.72
1	16	98570	89672	5264	3634	5716	208.76	69	2.52
1	17	106495	36630	59365	10500	13800	466.50	239	8.08
1	19	78179	67245	8386	2548	3376	155.46	73	3.36
2	1	117603	11052	97323	9228	12574	384.91	33	1.01
2	2	97167	86386	8630	2151	3510	130.04	40	1.48
2	3	97441	73835	19907	3699	4098	151.40	83	3.07
2	4	62202	53084	6629	2489	3334	192.96	37	2.14
2	5	155943	103683	44496	7764	10620	245.17	173	3.99
2	7	26367	21268	4199	900	1144	156.20	20	2.73
2	8	109307	97326	8730	3251	4774	157.23	59	1.94
2	9	44314	35014	7300	2000	2764	224.54	30	2.44
2	10	96153	76111	15709	4333	6120	229.13	74	2.77
2	11	89954	74901	11083	3970	5658	226.44	57	2.28
2	12	109649	100880	6480	2289	3120	102.44	55	1.81
2	13	194962	141931	39680	13351	17122	316.16	210	3.88
2	14	143040	128853	10583	3604	5060	127.35	83	2.09
2	15	29099	24209	3628	1262	1844	228.13	25	3.09
2	16	106895	86393	16377	4125	5092	171.49	65	2.19
2	17	47355	33583	13051	721	914	69.48	41	3.12
Mean		91106	70460	16855	3791	5243.09	200.90	78.36	3.09
Standard deviation		40877	35832	19545	2849	3753.44	82.94	55.19	1.46

Table B: Physical activity and sitting time data for valid data for included participants

building	participant	number of toilet trips	number of kitchen trips	number of trips to other desk areas	number of trips from desk	bathroom trips per hour	kitchen trips per hour	trips to other desks per hour	trips from desk per hour
1	1	6	12	11	21	0.42	0.84	0.77	1.48
1	2	12	42	67	76	0.42	1.47	2.34	2.66
1	3	11	12	18	34	0.36	0.39	0.59	1.11
1	4	13	39	45	57	0.57	1.72	1.99	2.52
1	5	11	65	51	89	0.30	1.80	1.41	2.47
1	6	21	19	25	49	0.57	0.52	0.68	1.33
1	7	3	8	9	13	0.38	1.01	1.14	1.65
1	8	2	5	7	9	0.28	0.71	0.99	1.28
1	9	13	18	7	41	0.83	1.15	0.45	2.62
1	10	4	11	9	21	0.35	0.97	0.79	1.85
1	11	0	18	44	42	0.00	0.79	1.93	1.85
1	13	8	18	23	39	0.28	0.62	0.80	1.35
1	14	1	42	46	54	0.03	1.36	1.49	1.75
1	15	7	11	36	20	0.18	0.28	0.92	0.51
1	16	20	12	16	40	0.73	0.44	0.58	1.46
1	17	18	84	78	125	0.61	2.84	2.64	4.23
1	19	4	34	24	52	0.18	1.57	1.11	2.39
2	1	6	56	43	51	0.18	1.71	1.32	1.56
2	2	13	19	7	31	0.48	0.70	0.26	1.15
2	3	19	21	8	40	0.70	0.78	0.30	1.48
2	4	7	12	16	27	0.41	0.69	0.93	1.56
2	5	20	38	83	87	0.46	0.88	1.92	2.01
2	7	2	8	0	12	0.27	1.09	0.00	1.64
2	8	6	30	15	40	0.20	0.99	0.49	1.32
2	9	9	24	30	19	0.73	1.95	2.44	1.54
2	10	16	32	33	45	0.60	1.20	1.24	1.68
2	11	18	22	15	46	0.72	0.88	0.60	1.84
2	12	13	19	6	43	0.43	0.62	0.20	1.41
2	13	26	62	73	114	0.48	1.14	1.35	2.11
2	14	21	32	4	56	0.53	0.81	0.10	1.41
2	15	3	3	2	9	0.37	0.37	0.25	1.11
2	16	4	36	51	46	0.13	1.21	1.72	1.55
2	17	2	6	4	8	0.15	0.46	0.30	0.61
Mean						0.40	1.03	1.03	1.71
Standard deviation						0.21	0.55	0.71	0.68

Table C: Trip data using valid data for included participants

C Additional figures

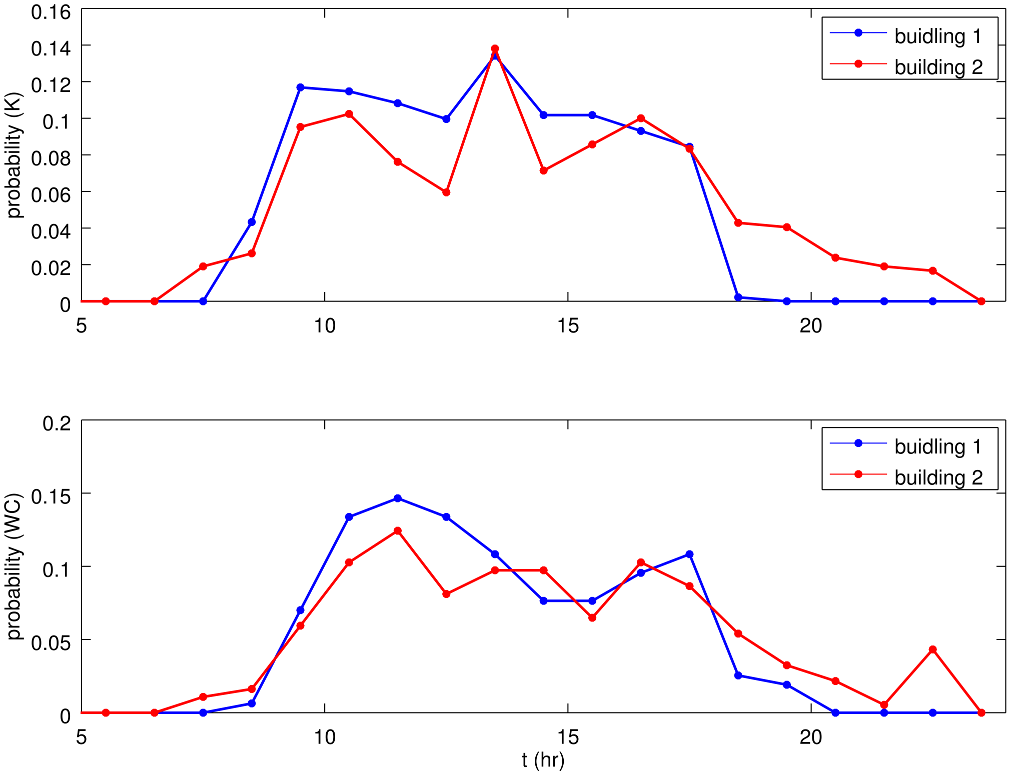

Figure A: Trip timing within the working day. Distribution of the time at which trips to kitchens and WCs occur for all participants within each building case study (n=33).

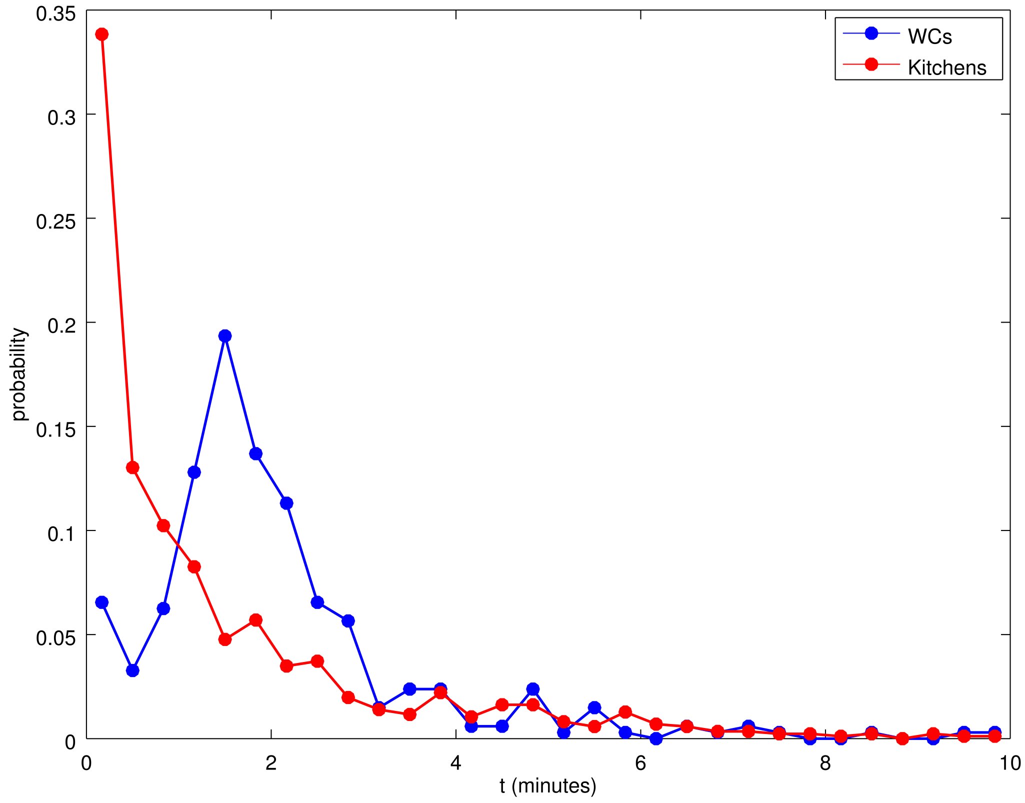

Figure B: Time spent at trip locations. Distribution of time spent in WCs and kitchens derived from all participant data across both case study buildings (n=33).

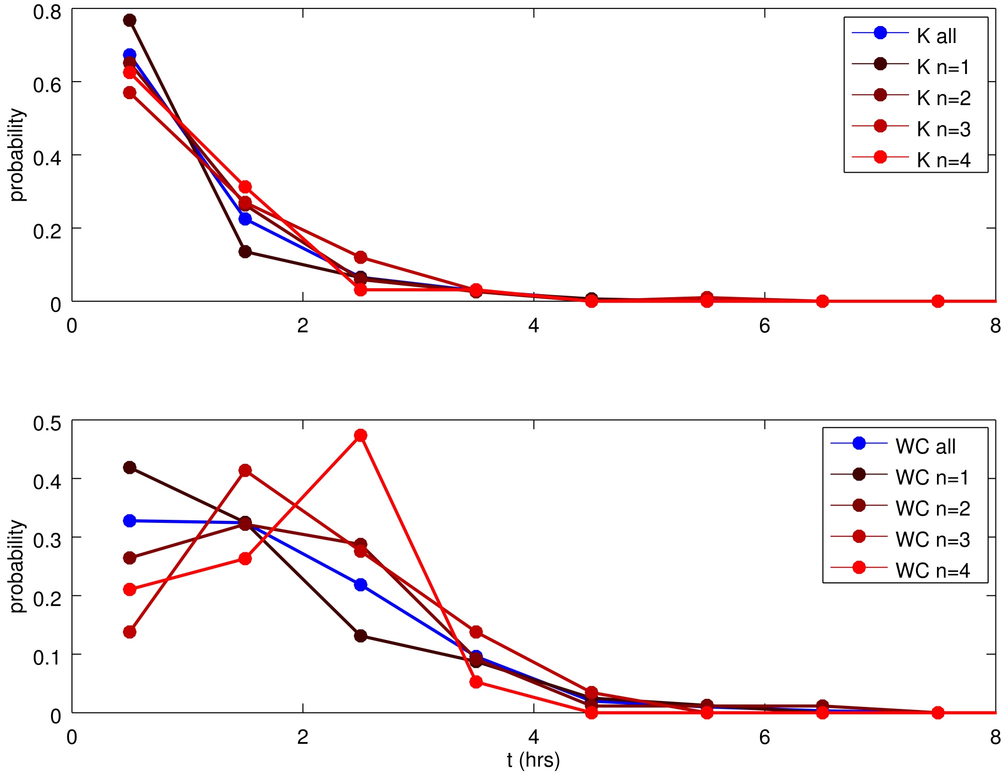

Figure C: Waiting time between trips. Distribution of time between trips to WCs and kitchens and between specific trip numbers derived from all participant data (n=33).
